# Study on the TOC concentration in raw water and HAAs in Tehran’s water treatment plant outlet

**DOI:** 10.1186/2052-336X-11-28

**Published:** 2013-11-12

**Authors:** Mahboobeh Ghoochani, Noushin Rastkari, Ramin Nabizadeh Nodehi, Amir Hossein Mahvi, Simin Nasseri, Shahrokh Nazmara

**Affiliations:** 1 Office of Environment and occupational Health, Ministry of Health and Medical Education, Tehran, Iran; 2 Center for Air Pollution Research (CAPR), Institute for Environmental Research (IER), Tehran University of Medical Sciences, Tehran, Iran; 3 Department of Environmental Health, School of Public Health, Tehran University of Medical Sciences, Tehran, Iran; 4 Center for Solid Waste Research (CSWR), Institute for Environmental Research, Tehran University of Medical Sciences, Tehran, Iran; 5 National Institute of Health Research, Tehran University of Medical Sciences, Tehran, Iran; 6 Center for Water Quality Research (CSWR), Institute for Environmental Research, Tehran University of Medical Sciences, Tehran, Iran

**Keywords:** Natural organic matter, Halo acetic acids, Seasonal variations, Tehran

## Abstract

A sampling has been undertaken to investigate the variation of haloacetic acids formation and nature organic matter through 81 samples were collected from three water treatment plant and three major rivers of Tehran Iran. Changes in the total organic matter (TOC), ultraviolet absorbance (UV254), specific ultraviolet absorbance (SUVA) were measured in raw water samples. Haloacetic acids concentrations were monitored using a new static headspace GC-ECD method without a manual pre-concentration in three water treatment plants. The average concentration of TOC and HAAs in three rivers and three water treatment plants in spring, summer and fall, were 4, 2.41 and 4.03 mg/L and 48.75, 43.79 and 51.07 μg/L respectively. Seasonal variation indicated that HAAs levels were much higher in spring and fall.

## Introduction

Chlorination is used in many countries to produce safe drinking water and decrease the incidence of water born infectious disease [1,2]. Disinfection by-products (DBPs)^a^ are formed due to reaction between disinfectant and natural organic matter (NOM)^b^ , such as humic acid and fulvic acid, which often can’t be removed during treatment process [3-5]. NOM is generated by physical, chemical and biological activities both in the watershed surrounding a water source and within the water source itself. NOM can be fractionated into hydrophobic and hydrophilic fractions [6]. Over 500 DBPs have been identified to date [7]. The most famous compounds include trihalomethanes, haloacetic acids, haloacetonitriles, haloaldehydes, chlorinated ketones, chlorophenols, chloropicrin [3,8]. Among these products THMs is important in the first rank, and then HAAs, due to their potential reproductive, carcinogenic and mutagenic effects. [7,9] HAAs are non-volatile, ionic and highly hydrophilic that includes nine compounds; chloroacetic acid (MCAA), dichloroacetic acid(DCAA), Trichloactic acid (TCAA), bromoactic acid (MBAA), dibromoacetic acid (DBAA), tribromoacetic acid (TBAA), bromochloroacetic acid (BCAA), dichlorobromoacetic acid (DCAA), dibromochloroacetic acid (DBCAA) [10]. Bromine acetic acids can be formed when disinfected water containing bromide ion [11]. There are numerous researches that showed the connection between these compounds with the occurrence of cancer, growth retardation, spontaneous abortion, and congenital cardiac defects [2]. Some studies showed that HAAs are more carcinogenic than THMs. DCAA was hepatoxic which promoted the cells accumulating the liver glycogen in rodents and produced neurotoxicity. Maternal rat fed with 2,730 ppm of TCAA in drinking water also demonstrated a significant increase in cardiac defects of fetus. Both DCAA and DBAA showed adverse male reproductive effects in animal studies. Some brominated-HAAs were also found inducing oxidative damages to DNA in the liver or they were toxic for cecal microbiota and mutagenic in the microsuspension assay. In some investigations, brominated-HAAs had slight or more significant adverse health effects than chlorinated-HAAs [12]. A recent study to the UK Drinking Water Inspectorate has proposed, HAAs should be classified as a “high priority” (the highest category in the list of regulatory chemical parameters to the routinely monitored) [7].

In response to the potential human health concerns of DBPs, many countries or international organizations have promulgated regulations to control THMs and HAAs in drinking water. United States Environmental Protection Agency (USEPA) according to the stage І DBPs has regulated maximum contaminate level (MCL) 60 μg/L for HAA5 (MCAA, DCAA, TCAA, MBAA, DBAA). This value was later reduced and recommended on stage ІІ to be 30 μg/L [9,13]. For protection of public health, World Health Organization (WHO) set the guidelines of MCAA, DCAA, and TCAA to be 20, 50 and 200 μg/L, respectively [14]. Due to the increasing concern on human health, particular attention has been recently paid to water treatment processes [2]. In Iran, chlorine gas is the most widely used primary disinfectant in water treatments, and HAAs and THMs are the most abundant DBPs (by weight) in drinking water. Currently, no national standard for HAAs in drinking water was established in Iran, only Institute of Standards and Industrial Research of Iran (ISIRI) has set MCLs for THMs [15]. Tehran is the capital of Iran. These days Tehran drinking water is provided mainly from surface and groundwater resources. Karaj, Jajrood and Lar are the three major rivers that provide water for 5 treatment plants. The water treatment plants 1, 2 (Jalalieh and Kan) are feed of Karaj River, 3–4 (Tehranpars) of Jajrood River and 5 (Sohanak) of Lar River. All these water treatments plants utilize chlorine gas for disinfection. No research has been done so far regarding the presence of HAAs in Iran drinking water. Therefore, the aim of this research was to determine the effective factor for the production of HAAs in raw water and measured their concentration in Tehran water treatment plant outlet.

## Material and methods

### Surface water sampling

Experiments were carried out on samples taken from three rivers that supply drinking water demand to Tehran city. Water samples were collected in the middle of the stream and at mid-depth and 2 times from each river. Water sampling was conducted monthly from April to December 2010. Samples were analyzed from the point of pH, Temperature, Total Organic Carbon (TOC) and UV-254 (UV absorbance at a wavelength of 254 nm). NOM content was characterized using two indicators, TOC and UV-254. Samples for measurement of these parameters were collected in 40 ml amber glass bottles. The pH and Temperature were measured at the sampling sites. Samples were carried to the laboratory, and stored in refrigerator at 4°C for the analysis of TOC, UV254 and Specific ultraviolet absorbance (SUVA). Total Organic Carbon (TOC) was determined by colorimetry (Method No.10129, low range (0.3-20 mg/L), DR/5000 device). The UV absorbance (UV-254) was analyzed in accordance with the standard method No.5910B (Ultraviolet Absorption Method) by using Lambda25 UV/Vis spectrophotometer. Specific UV absorbance (SUVA) (L/mg.m) was calculated as a ratio of UV absorbance at 254 nm (1/m) to DOC (mg/L). Potassium Hydrogen Biphthalate (KHP) was used to check the precision of the spectrophotometer [16]. Specific UV absorbance (SUVA) at 254 nm was used as an index of aromaticity contained in humic substances. Humic substances have grater aromatic carbon contents than non humic material [17]. SUVA is used to describe the composition of the water in terms of hydrophobicity and hydrophilicity. It has been considered that if SUVA is greater than 4 (SUVA > 4), NOM will be considered to have more hydrophobic (humic) fraction in nature and plays a major role in the formation of DBPs. And also if SUVA is less than 2 (SUVA < 2), hydrophilic (non-humic) substances are considered to play a major role in the formation of DBPs. If SUVA is between 2 and 4, water is mixture of hydrophobic and hydrophilic [13].

### Water sampling from treatment plant

Samples for HAAs analysis were taken from outlet of Jalalieh, Tehranpars and Sohanak treatment plants. These treatment plants provid parts of drinking water for Tehran city. Three samples were taken from the outlets at each sampling event. The samples were collected on the first Friday of every month from April to December 2010 (nine sampling events). Samples were analyzed from the point of pH, free residual chlorine and HAAs. The pH and free residual chlorine (mg/L) were measured in site sampling with phenol red and DPD tablets, respectively. For HAAs analysis, samples were stored in 40 ml amber glass container with screw capped and with PTFE-faced septa. Ammonium chloride was added to the samples bottles onsite as a dechlorinating agent to convert free chlorine to monochloramine, as monochloramine has less reactivity than free chlorine [18] also for avoiding DBPs formation during samples collection and transportation [12]. After water sampling, samples were kept in 4°C and shipped to the laboratory immediately and samples were refrigerated (4°C) until extraction.

### Glassware and reagents

Monochloroacetic acid (MCAA, 99%), Dichloroacetic acid (DCAA, ≥98%), Trichloroacetic acid (TCAA, 99.5%) were obtained from Merck company. Monobromoacetic acid (MBAA, 99%) and 2–3 Dibrompropionic acid (98%) are used as internal standard and were purchased from Sigma-Aldrich. Dibromoacetic acid (DBAA, ≥97%) was supplied by Fluca. The derivatization reagent dimethyl sulfate (DMS), as well as the ion-pairing agent tetrabutylammonium hydrogen sulfate (TBA-Hso4), dechlorinating agent, ammonium chloride and anhydrous sodium sulfate were obtained from Merck at high purity. TOC reagent (low range) was provided by Hach Lange. Water HPLC grade was purchased from Merck Company. Samples for measurement of HAAs were collected in 40 ml amber glass vials and were poured in 10 ml vials (specific vial GC). These vials were cleaned before sampling and analysis. At first it was rinsed with detergent and consecutively withdeionized water and secondly with 1/10 HCL/Water for 8 hours and then again with deionized water and finally with water HPLC grade and baked at 110°C overnight.

### HAAs analysis

Different methods can be used for analyzing HAAs. Method No.6251 Standard method is nowadays not commonly used in new research as it utilizes diazomethane. Recent researches use Method No.552.2, 552.3 EPA. However, in this paper us useda direct derivatization of HAAs by dimethyl sulfate to avoid the usual boring step, to reduce the analysis time and considering the importance of the concept of green chemistry in new research. HAAs were analyzed using a new static headspace GC-ECD method without a manual pre-concentration [19]. 5 mL water sample were taken into a 10 mL headspace vial without pre-filtration. After adding 5 g of pure Na_2_ SO_4_ (anhydrous), 800 μg tetrabutylammonium hydrogensulfate (TBA-HSO_4_), as aquatic solution and 100 μL dimethylsulfate, the headspace vial was closed by a gas-tight cap. The headspace vial was moved to a 60°C water bath, where the solution was mixed for 30 min with a glassy magnetic stirring bar, allowing in situ derivatization. After all these procedure the sample will be ready for analytical measuring in only a few minutes. All analysis for HAAs was carried out by a Varian cp-3800 gas chromatography with electron capture detection (GC-ECD). Separations were conducted on a fused-silica capillary column (50 m, 0.32 mm, 0.25 μm), with helium as a carrier gas, at a linear velocity of 35 ml/min and pressure 5 psi. Nitrogen was used as makeup gas. The instrumental temperatures were as follows: injector temperature, 275°C; initial oven temperature, 40°C (held for 3 min), increased to 50°C at a rate of 5°C min^-1^ (held for 2 min) increased to 110°C at a rate of 5°C min^-1^ (held for 2 min), then increased to the final temperature 250°C at a rate of 30°C min^-1^, where it was held for 1 min. The inlet was operated in split less mode and detector temperature was set at 300°C. The limits of detection at a signal-to-noise (S/N) ratio of 3 were 0.5 μgL^-1^. Mean concentration of HAAs among outlet water of treatment plants and in different months were analyzed with Excel and SPSS (linear regression) software.

## Results

Table 1 present the result of water quality measurement of raw waters from three rivers. As shown in Table 1, Total organic carbon (TOC) concentration of water samples from Lar River was 3.1 mg/L and in the range of 1.62-4.21 mg/L. And also the mean TOC concentration of water from Jajrood River was 3.87 mg/L and in the range of 2.72-4.69 mg/L. The result of Table 1 also indicated that the mean TOC concentration of Karaj River was 3.45 mg/L and in the range of 2.41-4.16 mg/L.

**Table 1:** The mean water quality parameters were measured in the three rivers

**River**	**Season**	**Temperature**	**pH**	TOC*	UV-254*	SUVA*
Karaj	Spring	9.33	7.5	3.99	0.17	4.32
	Summer	15.33	7.4	2.51	0.12	5.17
	Fall	11.66	7.4	3.80	0.15	4.05
Lar	Spring	10	7.8	3.60	0.16	4.49
	Summer	15.66	7.9	1.79	0.09	5.22
	Fall	11.83	7.4	3.90	0.15	4.07
Jajrood	Spring	9.96	7.3	4.42	0.18	4.18
	Summer	15.3	7.4	2.77	0.13	4.78
	Fall	11.33	7.3	4.42	0.18	4.06

*: TOC (mg/L), UV 254 (1/cm), SUVA: specific UV absorbance (L/mg m).

The result of SUVA measurements in three rivers was found to be greater than 4 (In this study TOC was used in calculating SUVA because the investigation water has very low turbidity, DOC representing 95% and more of TOC). Other characteristics of the three river’s water studied during this research are summarized in Table 1.

Table 2 present the result of HAAs measurement of outlet water from Jalalieh, Tehranpars and Sohanak treatment plants. As shown in Table 2, HAA concentration of water samples from the Jalalieh water treatment plant was 47.77 μg/L and in the range of 42.76-53.28 μg/L. And also the mean HAAs concentration of outlet water Tehranpars treatment plants was 54.66 μg/L and in the range of 47.87-59.84 μg/L. And also the mean HAAs concentration of the Sohanak treatment plant was 40.10 μg/L in the range of 32.99-45.48 μg/L. The other characteristic of the outlet water treatment plants studied during this research are summarized in Table 2.

**Table 2:** The mean water quality parameters were measured in outlet water treatment plants

**Treatment plant**	**Season**	**pH**	*Free residual chlorine	DCAA*	*TCAA	*Total HAAs
Jalalieh	Spring	7.59	0.87	37.93	11.09	49.01
	Summer	7.60	1.00	32.63	11.45	44.07
	Fall	7.67	0.87	38.28	11.94	50.22
Tehranpars	Spring	7.67	0.93	46.00	11.23	56.23
	Summer	7.53	1.00	37.04	11.76	48.83
	Fall	7.53	0.77	45.91	12.68	58.92
Sohanak	Spring	7.87	0.87	26.97	10.18	41.71
	Summer	7.87	0.87	23.59	10.91	34.50
	Fall	7.83	0.80	32.55	11.54	44.09

*: CL (mg/L); DCAA, TCAA and HAAs (μg/L).

## Discussion

The data presented in Table 1 show that SUVA in three rivers in spring, summer and fall was greater than 4; therefore, the fraction of hydrophobic NOM was higher than hydrophilic. Studies Kanokkantapong and et al. (2006) shown that hydrophilic neutral fraction in raw water in Bangkok, Thailand, was greater than hydrophobic [6]. Rodriguez et al. (2004) reported that SUVA in all of the seasons of 2004 year in Province of Qubec, Canada in raw water was greater than 4 and in treated water was less than 2 [20]. The studies of Zazouli et al. (2007) showed the DOC concentration of unfractionated water sample from the Lar, Karaj and Jajrood rivers was 8.53, 11.33 and 12.90 mg/L respectively. And also the SUVA in these three rivers was 2–4 and hydrophobic fraction was greater than hydrophilic [13]. Zazouli et al. (2007) reported that NOM concentration in Jalalieh and Tehranpars water treatment plants were 2.47 and 1.63 mg/L, respectively. And also fraction of hydrophobic NOM was higher than hydrophilic NOM [9]. Fearing et al. (2004) reported that DOC concentration in water at Albert water treatment works from the north of England is 7.8 to 11.2 mg/L. They were sampled at three different times during (April 2002 to March 2003) and as can be seen the nature of the water can vary throughout the year [21].

**Figure 1:**
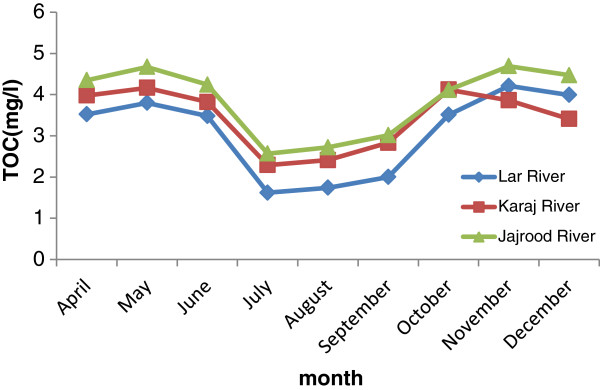
The month variation of TOC was indicated in three rivers.

Presence of NOMs as precursors forming DBPs is very important. The amount and type of these products are depending on the characteristics natural organic matter. In general the presence of total organic carbon and UV absorption at 254 nm in water, depending on water source, climate and seasons are different. Result showed that TOC and UV-254 concentration in raw waters were significantly higher during spring and fall and lower in summer. Figure 1 shows that this variation in three rivers. Accordingly, the trend for seasonal TOC variability was similar to study uyak et al. (2008). These observations indicate a shift not only in the quantity but also in the composition of NOM following precipitation and suggest that runoff leached humic substances from the upper soil layer. Soil and hydrology affect NOM, as hydrologic conditions define the flow paths that water takes in transporting DOC to surface water supplies like rivers and lakes, and interact with soil horizons of differing mineral and inorganic character [22].

**Figure 2:**
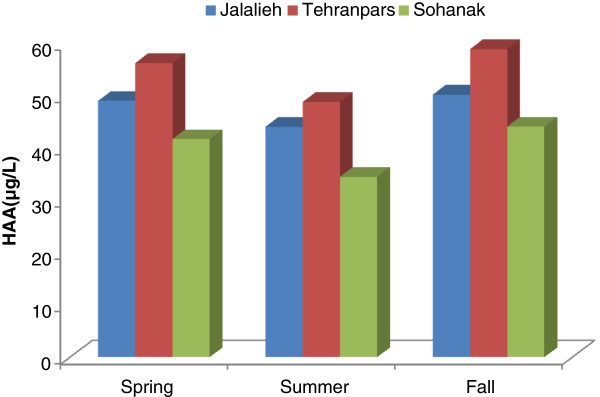
Seasonal variation of HAAs concentration was indicated in outlet water treatment plants.

The data presented in Table 2 show that HAAs concentration in outlet water treatment plants (Jalalieh, Tehranpars and Sohanak) in spring and fall is higher than summer. The higher HAAs concentrations during fall and spring could be explained by the greater presence of precursors favoring the formation of HAAs, because the highest average water TOC and SUVA values were also observed in two seasons. Figure 2 shows that this variation in three treatment plants. The result indicated that changing parameters such as pH, temperature and residual chlorine is not affecting the concentration HAAs, even though it can be said that these compounds can change in different seasons depending on TOC and UV-254. The Regression analysis results in the Karaj River shows that UV-254 and TOC are having great correlation with R^2^ = 0.86, sig <0.001, showing the existence of strong relation between these two parameters. Accordingly, the regression analysis of HAA and TOC also resulted in, R^2^ = 0.82, sig <0.001 and, that of Halo acetic acids and UV-254 is R^2^ =0.59, sig = 0.015. Therefore one can conclude that TOC can affect HAAs forming. The relationship between TOC and Halo acetic acids indicated that increasing TOC in the spring and fall lead to HAAs forming. The study of Nikolous et al. (2004) showed Seasonal variation water treatment plant in Athens for TTHMs and HAAs generally followed that of humic substances content with peaks occurring in autumn and spring [23]. Zhang et al. (2010) reported that the average total HAA concentrations in three water supply systems in England were approximately 65 and 85% higher in the fall than those in the spring and winter, respectively [24]. Uyak et al. (2008) reported the seasonal variation of DBPs in three major water treatment plants in Istanbul, Turkey and the highest average HAA concentrations were found in spring and fall. It was concluded that seasonal variations of DBP were related to changes in NOM quantity and characteristics of water sources. THMs and HAAs are produced as a result of reactions between chlorine and organic matters. A higher DOC level is thus likely to produce more THMs and HAAs [22].

During the period under study, MCAA, MBAA and DBAA were the products not detectable among the five HAAs in the measured samples (values lower than the detection limit) and this can be explained primarily by the very low levels of bromide ion in the water sources, which lead to a very low concentration of brominated CDBPs. DCAA and TCAA which are the major HAAs found in all samples.

The result of various studies showed that DCAA and TCAA are major HAA species [2,12,20,22,24-27]. In the study of Rodriguez et al. (2004) only DCAA and TCAA were observed. The result of Table 2 showed that the concentration of DCAA decreased in summer.

The study of Rodriguez et al. (2007) showed that the degradation of DCAA in summer was very probably due to the highly favorable conditions for microbial activity within the filter. In fact, Williams and Fauntleroy (2005) reported that specific type of bacteria (identified as a Burkholderia and Sphingomonas species) may degrade dihalogenated DBPs in warm waters [28,29].

## Conclusions

The highest total HAAs concentrations of outlet water treatment plants in Tehran is measured in this study and found to be below the MCL of 60 μg/L established by EPA. The DCAA and TCAA levels were also found to be below the WHO guideline (50 and 200 μg/L respectively). Nevertheless, the concentration of HAAs shown in water samples was close to MCL standard that can be a warning signal for authorities in the water industry to think about it. It is also essential that the necessary precautions be taken to remove TOC from the raw water before chlorination.

## Endnotes

^a^Disinfection By-Products.

^b^Natural Organic Matters.

## Abbreviations

DBPs: Disinfection by-products; NOM: Natural organic matters.

## Competing interests

The authors declare that they have no competing interesting.

## Authors’ contributions

This study is a part of a research project. The study was directed by Dr. AHM who is the corresponding author and made the final preparation of article. Engineer MG was engaged in sample preparations and laboratory work. Dr. NR, RN and SN helped on analytical consulting. Engineer SN as GC-ECD expert performed the experiments by this device. The overall implementation of this study including the design, sample collection and preparations, laboratory experiments, data analysis, and manuscript preparation was performed by the corresponding author and the above team. All the authors have made extensive contribution into the review and finalization of this manuscript. All authors have read and approved the final manuscript.
